# Supplementary material for: A novel approach to topological network analysis for the identification of metrics and signatures in non-small cell lung cancer
Source: Sci Rep. 2023 May 22;13:8223. doi: 10.1038/s41598-023-35165-w (PMC10202911; doi:10.1038/s41598-023-35165-w)
Supplement: Supplementary file 1 — Supplementary Information. [file 41598_2023_35165_MOESM1_ESM.pdf]

Supplementary Information

Isabella Wu and Xin Wang

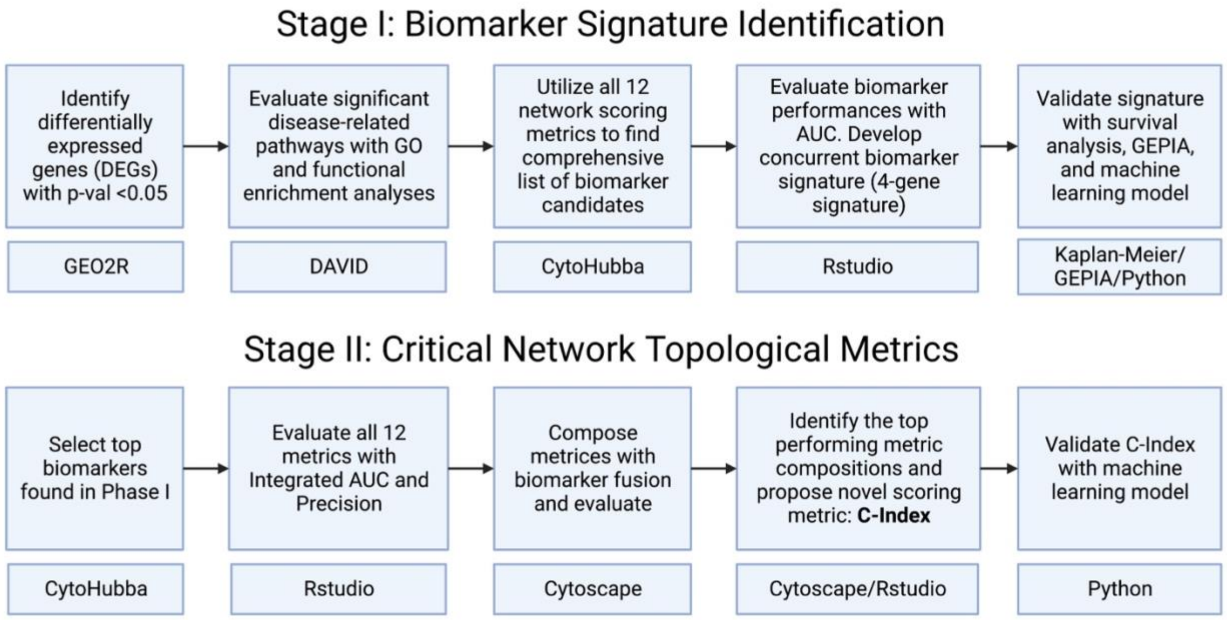

Supplementary Figure S1. Study overview with Stage I: Biomarker Signature Identification, and Stage II: Critical Network Topological Metrics

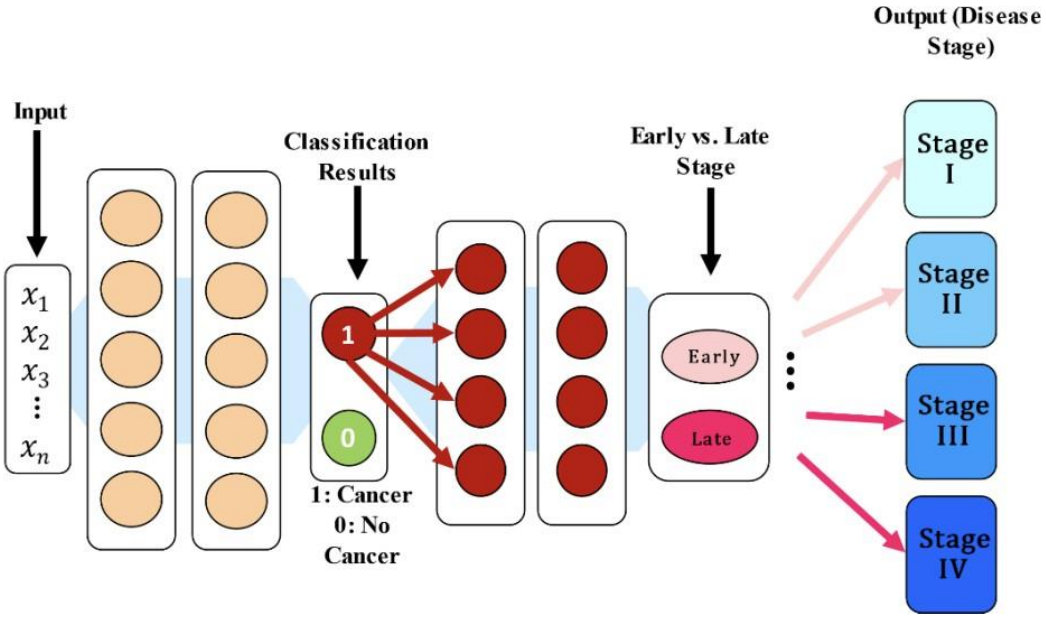

Supplementary Figure S2. Cascading model design

Supplementary Table S1. Summary of patient characteristics of three GEO datasets

GEO accession	GSE31210	GSE33356	GSE50081
**No. of patients (total)**	246	120	181
Health	20	60	0
Tumor	226	60	181
**Gender**			
Male	116	0	98
Female	130	120	83
**Histological type**			
LUAD	226	60	129
LUSC	0	0	52
**Stage**			
I	168	35	127
II	58	12	54
III/IV	0	13	0

Supplementary Table S2. AUC performances of biomarkers in the validation dataset

Biomarkers	Integrated AUC
AGER	0.9242
RASIP1	0.8695
CA4	0.9422
CAV1	0.9232
AGER+RASIP1+CA4+CAV1	0.9568
All Top 10	0.9358

Supplementary Table S3. AUC performances of scoring metrics in the validation dataset

Scoring metric	Integrated AUC
Degree	0.7368
Clustering Coefficient	0.9021
Bottleneck	0.9137
All Metrics	0.6600
C-Index	0.9221
